# Digital intervention increases influenza vaccination rates for people with diabetes in a decentralized randomized trial

**DOI:** 10.1038/s41746-021-00508-2

**Published:** 2021-09-17

**Authors:** J. L. Lee, L. Foschini, S. Kumar, J. Juusola, J. Liska, M. Mercer, C. Tai, R. Buzzetti, M. Clement, X. Cos, L. Ji, N. Kanumilli, D. Kerr, E. Montanya, D. Müller-Wieland, C. G. Ostenson, N. Skolnik, V. Woo, N. Burlet, M. Greenberg, S. I. Samson

**Affiliations:** 1 Evidation Health, San Mateo, CA USA; 2 Emory University, Atlanta, GA USA; 3 Sanofi, Gentilly, France; 4 Sanofi Pasteur, Swiftwater, PA USA; 5 Sapienza University of Rome, Rome, Italy; 6 University of British Columbia, Armstrong, British Columbia Canada; 7 Grup de Recerca Epidemiològica en Diabetis des de l’Atenció Primària (DAP-CAT) Group, Unitat de Suport a la Recerca Barcelona, Fundació Institut Universitari per a la recerca a l’Atenció Primària de Salut Jordi Gol i Gurina (IDIAPJGol), Barcelona, Spain; 8 Primary and Hospital Innovation Department, Innovation Office at Institut Català de la Salut, Barcelona, Spain; 9 Peking University People’s Hospital, Beijing, China; 10 Northenden Group Practice, Manchester, UK; 11 Sansum Diabetes Research Institute, Santa Barbara, CA USA; 12 Hospital Universitari Bellvitge-IDIBELL, CIBERDEM and University of Barcelona, Barcelona, Spain; 13 University Hospital RWTH Aachen, Aachen, Germany; 14 Karolinska Institute, Stockholm, Sweden; 15 Sidney Kimmel Medical College, Thomas Jefferson University, Philadelphia, PA USA; 16 University of Manitoba, Winnipeg, Canada; 17 Sanofi, Paris, France; 18 Kyowa Kirin International, Marlow, United Kingdom; 19 Sanofi Pasteur, Lyon, France

**Keywords:** Diabetes, Public health, Preventive medicine, Outcomes research

## Abstract

People with diabetes (PWD) have an increased risk of developing influenza-related complications, including pneumonia, abnormal glycemic events, and hospitalization. Annual influenza vaccination is recommended for PWD, but vaccination rates are suboptimal. The study aimed to increase influenza vaccination rate in people with self-reported diabetes. This study was a prospective, 1:1 randomized controlled trial of a 6-month Digital Diabetes Intervention in U.S. adults with diabetes. The intervention group received monthly messages through an online health platform. The control group received no intervention. Difference in self-reported vaccination rates was tested using multivariable logistic regression controlling for demographics and comorbidities. The study was registered at clinicaltrials.gov: NCT03870997. A total of 10,429 participants reported influenza vaccination status (5158 intervention, mean age (±SD) = 46.8 (11.1), 78.5% female; 5271 control, Mean age (±SD) = 46.7 (11.2), 79.4% female). After a 6-month intervention, 64.2% of the intervention arm reported influenza vaccination, vers us 61.1% in the control arm (diff = 3.1, RR = 1.05, 95% CI [1.02, 1.08], *p* = 0.0013, number needed to treat = 33 to obtain 1 additional vaccination). Completion of one or more intervention messages was associated with up to an 8% increase in vaccination rate (OR 1.27, 95% CI [1.17, 1.38], *p* < 0.0001). The intervention improved influenza vaccination rates in PWD, suggesting that leveraging new technology to deliver knowledge and information can improve influenza vaccination rates in high-risk populations to reduce public health burden of influenza. Rapid cycle innovation could maximize the effects of these digital interventions in the future with other populations and vaccines.

## Introduction

Seasonal influenza is associated with approximately 290,000–640,000 deaths worldwide each season^1^, and impacts approximately 21 million people in the United States annually, resulting in significant public health and economic burdens^2^. Preventing viral illnesses such as influenza is truly a global concern, given the potential for transmission in a modern, global culture, with this global risk and impact having been emphasized by the ongoing COVID-19 pandemic. People with diabetes (PWD), face increased risks from influenza, including poor glycemic control, pneumonia, premature death, acute cardiovascular complications, and hospitalizations^3–5^ which may result in a significant burden to the personal costs of healthcare for PWD. Vaccination remains the most effective primary prevention method against influenza, with effectiveness ranging from 29 to 48%^6,7^. Vaccination for influenza in PWD is effective in reducing the risk of hospitalizations and mortality^3,8^, as well as the overall cost of hospitalizations^3,7^. Influenza vaccination has also been shown to be safe for PWD and does not impact an individual’s ability to engage in daily activities in the days following vaccination^5,7,9^. However, vaccination rates remain suboptimal, consistently falling under the 70% vaccination rate goal set by national guidelines for all individuals in the United States^10^. In 2015 in the United States, 61.6% of adults with diabetes received an influenza vaccine^11^. During 2016–17, national rates of influenza vaccination were approximately 40% in adults without any high risk conditions, and 59.7% for adults with a variety of high risk conditions (including diabetes)^12^.

Therefore there is a need for effective and scalable solutions to increase influenza vaccination rates in PWD. While a number of randomized controlled trials (RCTs) have assessed the effectiveness of interventions for increasing influenza vaccination rates, many have focused on other age groups or populations^13^. One prospective digital interventional study demonstrated the potential effectiveness of general messaging and incentives via a health-related smartphone application (app) to increase vaccine uptake in a general Canadian population^14^, suggesting this kind of intervention could be effective in PWD. A large RCT using digital messaging was also effective in increasing vaccination rates in the general population of adults in the United States^15^. The use of health information technology (e.g., searching the internet for health information, emailing providers) and even simple electronic reminders delivered via digital patient portals have resulted in increased influenza vaccination rates, suggesting the potential of simple digital solutions^16,17^. One of the primary reasons PWD report not getting vaccinated is a belief that they are not in a high-risk group, and providing education on the increased risk of negative health outcomes following influenza infection has shown promise in increasing vaccination rates in other populations^18^. Additional reasons include fear of adverse reactions, difficulties with accessing the vaccine (e.g., time, health center access), or other beliefs surrounding the influenza vaccine (e.g., not effective, transmits the flu)^19^. Therefore, digital messaging that counters this lack of knowledge and barriers to vaccination could be effective for increasing uptake.

The aim of this study was to evaluate the effectiveness of a digitally administered intervention to increase influenza vaccine rates for PWD using a decentralized, blinded RCT. The primary endpoint was to examine the difference in self-reported influenza vaccination rates in 2 groups: PWD who received a digital intervention (PWD-I) and PWD who received no intervention (PWD-C). The following exploratory associations were also examined: (a) the impact of engagement with interventions on the influenza vaccination; (b) the impact of the timing of the intervention messages during influenza season on influenza vaccination status; (c) the reported level of influence on getting the influenza vaccine by each intervention message type within the PWD-I group; (d) the level of engagement with each intervention message within the PWD-I group; and (e) the impact of a healthcare worker’s recommendation on getting the influenza vaccine.

## Results

### Participant description

**Fig. 1:**
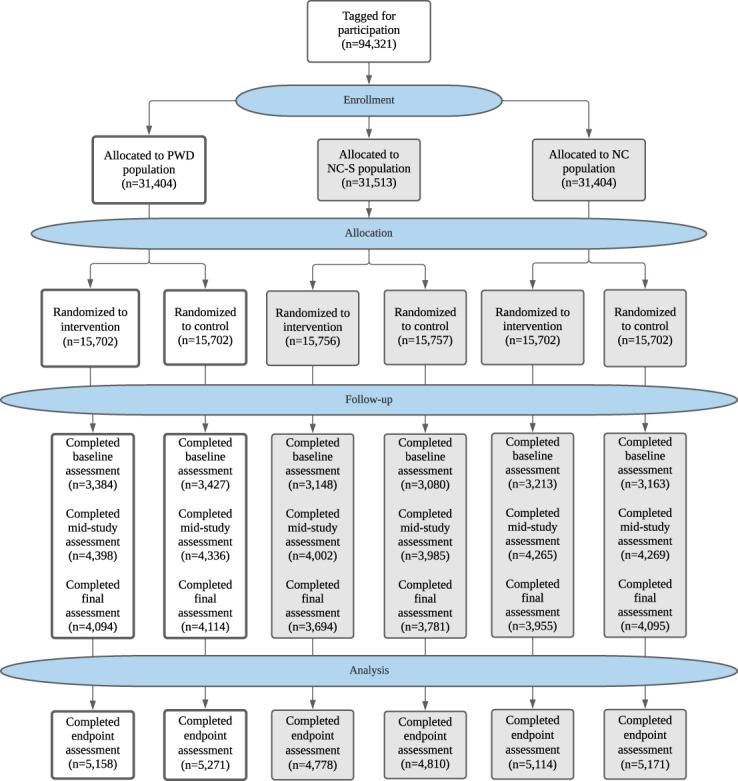
CONSORT flow diagram. CONSORT diagram presents the study flow for the larger investigation (NCT03870997). PWD people with diabetes, NC-S normal controls-similar, NC normal controls.

**Fig. 2:**
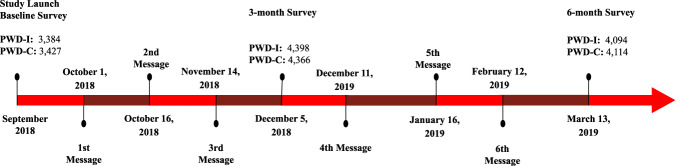
Study flow and intervention delivery. Depicts the study flow for participants and the timing of the intervention messages. The timing of the surveys and the corresponding *n* participants who completed those surveys is also included. PWD-I People with diabetes-intervention group, PWD-C People with diabetes-control group.

The study was launched in September of 2018 and the last participant completed the final survey in April of 2019, with the intent to capture outcomes during the 2018–2019 influenza season in the United States. A CONSORT diagram is included (Fig. 1) as a description of the larger investigation, including information on the 3 cohorts. For the PWD cohort, a total of 31,404 individuals were randomized, resulting in 15,702 in the PWD-I group and 15,702 in the PWD-C group. The PWD-I group consisted of 5158 individuals who completed the mid-study or final survey reporting their influenza vaccination status, and 5271 in the PWD-C group who reported their influenza vaccination status over the same interval. Approximately one-third of participants who were enrolled and randomized reported on the final endpoint. Figure 2 shows the flow of participants through the study, timing of the surveys, and timing of the intervention messages.

Descriptive characteristics for the sample are presented in Table 1. Demographic variables of age, race/ethnicity, and sex were not different between the two groups, indicating that randomization was successful. The participants were predominantly female, White, and in the middle age range and had high levels of income and education. Hypertension, depression, and high cholesterol were the most commonly reported comorbidities, with a small proportion reporting coronary artery disease. Participants included individuals from 48 of the United States, as well as the District of Columbia, United States Armed Forces, and Guam. A total of 101 individuals reported discrepant answers at the 3-month and 6-month questionnaires with regard to vaccination status. The effect size remained unchanged and statistically significant after re-running the primary outcome without these individuals.

**Table 1:** Participant demographics.

Sample that reported on primary endpoint of vaccination status			
Age (in years)		PWD: intervention (*N* = 5158)	PWD: Control (*N* = 5271)
		Mean (SD)	Mean (SD)
		46.8 (11.1)	46.7 (11.2)
		*n* (%)	*n* (%)
Sex	Female	4048 (78.5)	4183 (79.4)
	Male	1096 (21.2)	1066 (20.2)
	Other	14 (0.3)	22 (0.4)
Race/ethnicity	African American/Black	468 (9.1)	451 (8.6)
	American Indian or Alaska Native	50 (1.0)	53 (1.0)
	Asian	163 (3.2)	169 (3.2)
	Caucasian/White	3990 (77.4)	4141 (78.6)
	Native Hawaiian or other Pacific Islander	19 (0.4)	18 (0.3)
	Other	137 (2.7)	134 (2.5)
	Multiracial	331 (6.4)	305 (5.8)
Ethnicity	Hispanic/Latinx	496 (9.6)	519 (9.8)
	Not Hispanic/Latinx	4662 (90.4)	4752 (90.2)
Participants that completed the baseline assessment			
		PWD: Intervention (*n* = 3384)	PWD: Control (*n* = 3427)
		*n* (%)	*n* (%)
Income	Less than $25,000	409 (12.1)	376 (11.0)
	$25,000–$34,999	343 (10.1)	381 (11.1)
	$35,000–$49,999	480 (14.2)	485 (14.2)
	$50,000–$74,999	679 (20.1)	729 (21.3)
	$75,000–$99,999	504 (14.9)	493 (14.4)
	$100,000–$149,999	439 (13.0)	437 (12.8)
	$150,000 or more	195 (5.8)	174 (5.1)
	I prefer not to answer	335 (9.9)	352 (10.3)
Education	Did not complete high school, no diploma	42 (1.2)	38 (1.1)
	Trade/technical/vocational training	258 (7.6)	251 (7.3)
	High school graduate or the equivalent	347 (10.3)	399 (11.6)
	Some college, no degree	779 (23.0)	795 (23.2)
	College graduate	1364 (40.3)	1328 (38.8)
	Graduate degree	504 (14.9)	517 (15.1)
	Doctorate degree	72 (2.1)	80 (2.3)
	I prefer not to answer	18 (0.5)	19 (0.6)
Medical comorbidities	Cancer	329 (6.4)	335 (6.4)
	Chronic heart failure	56 (1.1)	57 (1.1)
	Coronary artery disease	97 (1.9)	112 (2.1)
	Depression	1988 (38.5)	2038 (38.7)
	High blood pressure	2318 (44.9)	2307 (43.8)
	High cholesterol	1914 (37.1)	1883 (35.7)
		M (SD)	M(SD)
	Body mass index (BMI)	33.3 (8.1)	33.3 (8.1)

*Note*. For BMI, 5 individuals in each cohort provided insufficient data to calculate BMI and are thus not included in analysis.

### Primary endpoint

With regards to the primary outcome, 3310 (64.2%) of 5158 participants from PWD-I reported flu vaccination, compared with 3220 (61.1%) of 5271 among the PWD-C participants, with an absolute intervention difference of 3.1%. The number needed to treat or message for this effect is 33 people to result in 1 additional influenza vaccination. After adjusting for age, sex, race, and comorbidities, the intervention group was more likely (RR 1.05, 95% CI [1.02, 1.08], *p* = 0·0013) to get flu vaccination than the control group.

### Exploratory outcomes

**Fig. 3:**
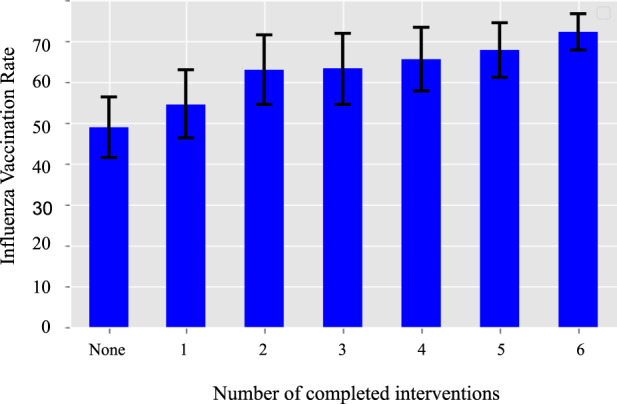
Vaccination rate per completed interventions for people with diabetes in the intervention group. Depicts the influenza vaccination rate for the PWD-I People with diabetes-intervention group, based upon the number of the intervention messages that were completed during the intervention period with standard error.

Greater rates of engagement measured by larger number of interventions completed was associated with an increased vaccination rate for PWD-I (Fig. 3). Participants in the PWD-I arm who completed at least one digital intervention were more likely to report influenza vaccination (PWD-I: 66.6%; PWD-C: 61.1%, OR 1.27 95% CI [1.17, 1.38], *p* < 0·0001) than PWD-C. Participants in PWD-I who responded to at least 3 messages had an even greater odds of reporting influenza vaccinations (PWD-I: 69.0%; PWD-C: 61.1%, OR 1.42, 95% CI [1.30, 1.56], *p* < 0·0001) than PWD-C. Of note, within PWD-I, participants who did not complete any messaging interventions were less likely to report influenza vaccination (OR 0.61, 95% CI [0.52, 0.72]) than those who completed interventions.

**Fig. 4:**
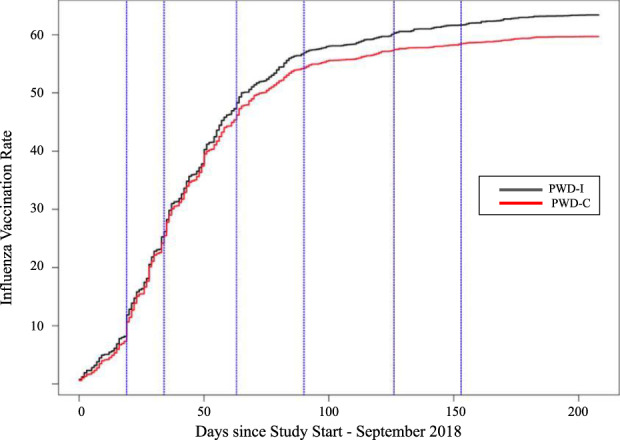
Time to vaccination in the PWD-I group compared to the PWD-C group. Depicts the vaccination rate over the course of the study using a Kaplan–Meier curve. PWD-I People with diabetes-intervention group; PWD-C People with diabetes-Control group.

**Fig. 5:**
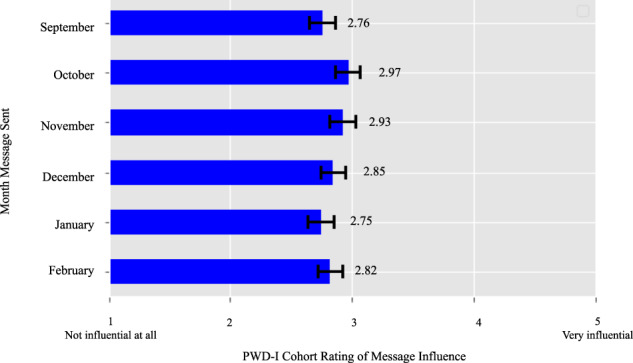
Level of influence from messages on getting the influenza vaccine. Depicts the ratings provided by the PWD-I: People with diabetes-intervention group regarding how influential each message was on their decision to get the influenza vaccine. Ratings include standard error and were not statistically significantly different between interventions.

**Fig. 6:**
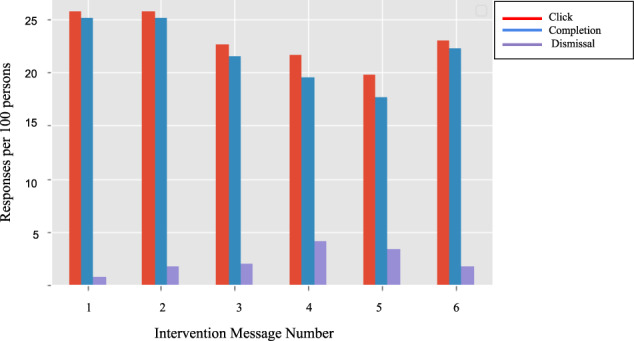
Different response types among PWD-I for each intervention message. Depicts the levels of engagement and different response rates for each intervention from the PWD-I: People with diabetes-intervention group.

The impact of timing of the intervention messages during influenza season on influenza vaccination rates within the PWD-I group, compared to the passage of time in the PWD-C group, is shown in the Kaplan–Meir curves in Fig. 4. Participants were asked to score the level of influence each message had on their decision to vaccinate (Fig. 5). Participants within PWD-I did not report differences in the effect of the messaging itself on the decision to vaccinate. Participants reported a mean score ranging between 2.75 to 2.93 for each of the messages. Figure 6 presents the response rates (i.e., number of responses per 100 persons) for each of the 6 different interventions. Each intervention message had a pattern of similar rates of clicks and completions with a much smaller rate of dismissals. Participants were most likely to respond to the first two messages with an overall decline in response rates in later months. Advice given by a healthcare worker to vaccinate had a strong effect on the decision to vaccinate among both control and intervention groups (OR 12.3, 95% CI [10.2, 15]). Participants who reported specific counseling to get influenza vaccination were slightly older than those who did not (44.9 vs 46.7 yrs., OR 1.01, 95% CI [1.01, 1.02], *p* value <0.001), more likely to have a self-reported cholesterol disorder (33.3 vs 45.1%, OR 1.18 95% CI [1.05, 1.32], *p* value <0.01).

## Discussion

This RCT used a digital intervention to significantly increase influenza vaccination rates in a large sample of PWD, reaching the primary endpoint with an increase of 3.1%. Influenza vaccination rates in the control group were consistent with those most recently published at a national population level for PWD^11^. Greater rates of engagement with a larger number of interventions were associated with increased vaccination rate, up to 8% for at least 1 or more intervention messages completed compared to the control group. Engagement rates with the intervention were overall high, with completion rates between 17 and 25% across intervention messages, consistent with digital communication industry standards^20^. The increase in vaccination rate seen in this study is much higher than that previously seen in other general digital interventions for increasing influenza vaccination rates^15^; it was, however, smaller than that was seen in a prospective interventional study (5% increase) that provided compensation for vaccination^14^. Although compensation was provided for completion of the interventions, the total compensation was only $0.30 per participant, suggesting that this was not a motivating factor for completing the intervention. The Kaplan–Meier curves demonstrate that from the start of the influenza season in 2018, the steepest rates of influenza vaccination took place in the first two months, with individuals in the PWD-I group being more likely to vaccinate earlier in the influenza season. Divergence between the two groups appeared to occur shortly after the first two months, with the PWD-I group continuing to experience more vaccinations until the fourth month, when both groups started to plateau. Given that the vaccination rates between the intervention and control groups diverged during the later months, the messages in months 3–6 might have had a greater impact on vaccination rates at an earlier date in the flu season. Although not statistically significant in difference, the two messages rated as directionally most influential for getting an influenza shot were in October and November, which were messages that targeted information on “The Flu and Diabetes” and centered around “World Diabetes Day,” respectively, suggesting a synergy could be possible between relevant disease-focused events and intervention deployments. These also provided information that was very specific to the diagnosis of diabetes and the impact of influenza. Results also indicated that people who received the recommendation from a healthcare worker to get vaccinated were vaccinated at greater rates, consistent with prior literature suggesting the importance of the role of the healthcare provider in promoting influenza vaccination^19^.

The analysis found that vaccination rates increased 3.1% in the intervention group compared to controls, but the association of completing the interventions with vaccination rate could be up to 8% for those who completed interventions. The CDC^21^ estimates that there are 26.8 million individuals with diabetes living in the US. An absolute increase of 3.1% in vaccination rate at a national level could represent approximately 830,000 additional PWD in the United States receiving the influenza vaccination. While there are unique characteristics in the medical and insurance system in the United States that present barriers to influenza vaccination that may not be encountered in countries with universal healthcare, suboptimal vaccination rates are a global concern, and this intervention could have broader generalizability. Strengths of the study include the large and diverse sample of PWD, representing most of the geographic United States with wide age and socioeconomic range. Additionally, a unique strength of a web-based RCT is the ability to blind participants not only to their treatment condition, but to their study participation, providing the opportunity to test how individuals would respond to the intervention in real-world settings. This clinical trial also enrolled participants quickly with high geographic variability, a strength of this decentralized trial format and consistent with other decentralized clinical trials^22^. As only approximately 33% of the participants completed the mid-point or final survey containing the primary endpoint, these results could be an inaccurate estimate of the true effect of the intervention given missing data. What remains true is that participants who completed 50% or more of intervention messages in the PWD-I cohort had an even higher vaccination rate than those who did not, suggesting that the current results could be an underestimate of the effect measured in this population. While self-report of influenza vaccination has shown good specificity in large populations, the most accurate estimate of vaccination status would result from a combination of self-report and medical claims data^23,24^. Future research should consider this approach to reduce potential self-report bias.

The primary shortcoming of this study is the limited generalizability of the study population which may not represent the entire United States population with diabetes. The majority of participants were female and of non-Hispanic white race/ethnicity. They also represented an educated population, with most having a college degree and above average income levels. It is also possible that the Achievement population represents a more digitally connected population, representative of those who seek out digital health resources. While the initial results from evaluating the Digital Diabetes Intervention are promising and demonstrated an effect in this sample, these results are not easily generalizable to a broader population of PWD. This study should serve as a building block for future research. The intervention content would benefit from further development and tailoring to reach individuals from diverse backgrounds effectively. Brewer and colleagues^25^ highlight the potential of mobile health interventions for improving health outcomes in individuals from diverse backgrounds, demonstrating that more individuals from racial and ethnic minorities use mobile apps and smartphones to access health information than white individuals and are generally receptive to participating in digital health research. Digital interventions have great potential for reducing health disparities if barriers to health equity surrounding internet connectivity and equitable access to hardware are reduced^26^. While delivered via the Achievement app, the intervention messages within the Digital Diabetes Intervention were built as individual webpages. For dissemination, messages like these could also be sent using hyperlinks via text or even as healthcare medical record portal messages. The COVID-19 pandemic has demonstrated the significant reach of digital health technologies, with increased adoption of telehealth services during the pandemic around the world^25,27^, highlighting the ability of individuals to adapt to changing models of healthcare. Approximately 33–52% of PWD use mobile apps to manage their health^28^, suggesting that a digital intervention may be especially impactful for PWD, with the increasing prevalence of app-connected continuous glucose monitors and the use of technology for managing diabetes.

This study shows that a relatively low-cost digital intervention using information from existing trusted sources is an effective way to increase vaccination rates among individuals with diabetes. Total compensation for the study, had all individuals completed all of the interventions, would have been approximately $4700, and recruitment leveraged an existing community of PWD, a generalizable cost-effective recruitment strategy for connected patient-focused or healthcare organizations. Digital interventions such as this have wider public health implications. A single influenza season costs the United States approximately $3.2 billion in medical costs, $8.0 billion in indirect costs, and $11.2 billion in overall economic burden^2^. There is also evidence that hospitalization due to influenza is 78% lower in PWD who are vaccinated than in PWD who are not vaccinated^3^, compared to a 58% lower chance of hospitalization in vaccinated people without diabetes. This suggests that high healthcare costs from hospitalization due to influenza are likely to impact this high-risk population of PWD disproportionately and could be reduced by vaccination. Further analyses should be conducted to understand how to maximize the cost-effectiveness of a digital intervention, compared to traditional in-person interventions. It would also be beneficial to evaluate efficacy of a digital intervention in increasing vaccination rates for individuals with other high-risk conditions, such as cardiovascular disease. As the world undergoes the most extensive vaccination campaign in history for the COVID-19 vaccine, the effectiveness of interventions that address hesitancy and misinformation surrounding vaccination is critical given the already high levels of hesitancy and resistance to vaccination^29,30^. This study supports that personalized and timely digital messages could make a significant difference in the number of people who get vaccinated. Although this intervention was developed with the input of patients via focus groups and expert opinions, the exact mechanisms of action for the intervention messages (i.e., the active ingredients) are unknown. Future research should aim to determine the mechanisms of action for digital interventions, as well as the motivations for initial and sustained engagement in the intervention. Obtaining this knowledge would help to generalize and apply this intervention to other populations to increase the reach of digital interventions for improving influenza vaccination rates.

## Methods

### Recruitment and enrollment

This decentralized RCT was conducted in the United States using the Achievement platform, an online study platform with more than 3.5 million members (myachievement.com, Evidation Health, Inc., San Mateo, CA) with a research community of users ranging in age from 18 to over 90 years, some with self-reported medical conditions. Achievement members can connect their activity trackers, fitness, and health apps to the platform and members accumulate points that are redeemable for monetary rewards for completing tasks. This study is a part of a larger investigation into the effectiveness of digital interventions for increasing vaccination rates, registered at clinicaltrials.gov, posted 12 March, 2019 (NCT03870997). The larger investigation consists of 3 cohorts, with 2 arms in each cohort (Fig. 1—CONSORT diagram). The 3 cohorts in the larger investigation are: (a) Individuals with self-reported diabetes (PWD cohort in white on Fig. 1), (b) individuals without self-reported diabetes but who are otherwise similar to PWD based on their demographic and activity tracker data (NC-S: normal controls who are similar) and (c) individuals without self-reported diabetes who are not in NC-S, but are similar to PWD with regards to age and gender (NC: normal controls). The focus of this presentation is on the particularly vulnerable population of PWD. Study design and sample size calculations were based upon this cohort. The study was a 6-month RCT, consisting of 2 arms: PWD who received the digital intervention (PWD-I) and PWD who received no intervention, serving as a control (PWD-C). Examples of the interventions are shown in the Supplementary Note 1.

Participants were blinded to study participation status, providing a pragmatic trial design that has been advocated for in order to enhance the generalizability and applicability of research conducted involving mobile health technologies^31^. This study was approved by the ethical committee, Solutions Institutional Review Board (IRB-Little Rock, AR) prior to any participant engagement. It was determined that participants faced no more than minimal risks from the study, as the intervention messages were consistent with current standard of care, and a waiver of informed consent was obtained from the IRB. Participants were informed that their survey responses and behavioral data would be used for research purposes via a Data Usage and Permissions Agreement at the beginning of each survey.

For inclusion in the study and allocation to the PWD cohort, participants needed to be at least 18 years of age, have self-reported type 1 or type 2 diabetes designated in their Achievement health profile, and live in the United States. For recruitment, a set of existing Achievement members who met the inclusion criteria were tagged for study inclusion. This constituted the set of study participants; they did not take any active steps to enroll in the study. These study participants were sent offers to receive “points” as compensation for completing surveys and study tasks, which is a routine component of participating in the Achievement platform. Total compensation for completing study surveys was the equivalent of $0.90, and total compensation for interacting with the six monthly Diabetes Digital Interventions was the equivalent of $0.30. Participants were randomized to the intervention (PWD-I) or control (PWD-C) arm prior to receiving any study offers using stratified randomization based upon sex and age as covariates with simple random sampling based upon an a priori list of participants. Due to the web-based nature of the study, blinding by investigator was not necessary as the messages and study experience were determined prior to enrollment in the trial, with the messages dispatched on predetermined dates without any additional investigator interaction. Participants were blinded not only to their intervention or control arm status, but also to the fact that they were enrolled in a study, a factor that is unique to a web-based RCT. Due to the study design and IRB determination that the study posed no more than minimal risk, information on adverse events was not solicited and none were spontaneously reported.

### Measures

Participants were sent an online baseline questionnaire, a mid-study assessment at three months, and a final assessment at six months. Due to the study design, participants could complete any or all assessments; completion of mid-study and final assessment was not predicated on completion of the baseline assessment. The primary endpoint of influenza vaccination status was collected in the three- and/or six-month questionnaires. Questions on demographics, influenza vaccination status, and healthcare worker recommendation were asked of all participants. Participants in the PWD-I cohort were additionally asked about their perceptions of the interventions, and their engagement with intervention tasks was assessed.

A demographic questionnaire was administered at baseline, three and six months to all participants. Age, sex, and race/ethnicity were collected in all three questionnaires. Participants were able to “select all” for race/ethnicity and comorbidities. Information on medical comorbidities, income, and education was collected only in the three- and six-month questionnaires. For assessing influenza vaccination status, all participants were asked, “Did you get vaccinated against the flu (sometimes called getting a flu shot) this season? This year’s flu season began in September 2018,” and responded with Yes/No. Self-reported influenza vaccination status has been shown to have good specificity and positive predictive value^23,24^. Influenza vaccination status was collected both at mid-study and end of study to minimize recall bias. Participants were also asked for the approximate date on which they received their influenza vaccine. All participants were asked in the final survey “Have any of your healthcare providers recommended that you get a flu shot?” and asked to indicate Yes/No. The PWD-I cohort was also asked about their perceptions of the interventions and how much each message-type influenced whether they got their flu shot, rating each message from 1—“not influential at all” to 5—“very influential.” For each intervention, engagement statistics were tracked for the PWD-I participants who (a) clicked to open the digital intervention, (b) continued to complete the call to action or (c) explicitly dismissed it.

### Intervention content

Each of the six monthly messages was structured in two parts: educational content, and a call to action for the participant to complete (Table 2). Education and recommendations provided in the intervention were based upon data from the United States Centers for Disease Control and Prevention (CDC), World Health Organization (WHO), International Diabetes Federation (IDF), and the American Diabetes Association (ADA), thus reflecting the current standard of care recommendations for PWD. Content was reviewed by an expert advisory board and focus group of PWD. Messages were communicated via the Achievement app using month-specific messages, for example occurring around World Diabetes Day in November and National Heart Month in February. Calls to action would award points to the participant for completing actions such as needing to find the nearest clinic offering flu shots (CDC Flu Finder Widget) or planning prompts^32^, which have been shown to be effective at increasing influenza vaccination^33^. Incentives (via Achievement points) were provided upon the completion of the call to action as described.

**Table 2:** Description of intervention messages.

Month	Educational message	Call to action	Goal of message
1	General influenza and vaccination facts^8^	Interactive quiz assessing knowledge	Education
2	Unique risks of flu-related complications for PWD^5,35^	Commitment to receive vaccine on selected date	Education, planning, and commitment
3	World Diabetes Day and information on flu-related complications for PWD^8^	Agree to be emailed more information on influenza vaccination	Education
4	Consequences of influenza, including decreased activity and steps/points^5^	Put in zip code to find clinic near them using vaccinefinder.org	Loss-aversion for points and location-aware message
5	Safety of vaccination and common side effects^36^	Put in zip code to find clinic near them using vaccinefinder.org	Decrease hesitancy and fear
6	Relationship between influenza and cardiac events for PWD^8,37^	Pledge to get flu shot on specific date during that month	Planning and commitment, education

Note. All intervention messages contained some information from publicly available materials from CDC, WHO, IDF, and ADA. Unique sources per-intervention are referenced above.

### Sample size calculations and analytical plan

For sample size determination, we estimated a 2.7% increase in vaccination rate between the PWD-I and PWD-C groups. This value was selected to be consistent with prior research and clinically meaningful^14,15^. Power analysis indicated the need for an analysis set of 4043 individuals in each arm of the study (total *N* = 8086) to achieve 80% power to detect a 2.7% increase in vaccination rate with a type I error rate of 0.05^34^. To account for potential non-response to study surveys, we tagged 31,404 PWD for study inclusion, with 15,702 randomized to each of the two arms (~25% assumed response rate).

Logistic regression models were used to compare self-reported vaccination rates in both study arms (PWD-I versus PWD-C) controlling for demographics (i.e., age, sex, and race/ethnicity) and comorbid conditions, to calculate the relative risk and absolute risk change using intent to treat analyses. Per protocol analyses were used on all other analyses. Kaplan–Meier plots were constructed to evaluate self-reported time-to-vaccination for the two arms. Logistic regression models were constructed to examine the effect of the number of completed interventions and recommendations given by health care workers on vaccination rates, controlling for demographics and comorbid conditions. Significance is reported at a type I error rate of 0.05, with a type II error rate of 0.20.

### Reporting summary

Further information on research design is available in the Nature Research Reporting Summary linked to this article.

## Supplementary information

Supplementary Information

Reporting Summary

## Data Availability

Qualified researchers may request access to the aggregate results and related study documents including the study report, study protocol with any amendments, blank case report form, statistical analysis plan, and dataset specifications. Further details on Sanofi’s data sharing criteria, eligible studies, and process for requesting access can be found at https://www.clinicalstudydatarequest.com.

## Code availability

The code that supports the findings of this study are available from the corresponding author upon reasonable request. Analysis to process and analyze data was generated with Python 3 and the R programming language.
