# Supplementary material for: Digital intervention increases influenza vaccination rates for people with diabetes in a decentralized randomized trial
Source: NPJ Digit Med. 2021 Sep 17;4:138. doi: 10.1038/s41746-021-00508-2 (PMC8448887; doi:10.1038/s41746-021-00508-2)

Supplementary Note 1

Digital Diabetes Interventions: Examples of messages sent to participants in the PWD-I cohort:
a) First message:

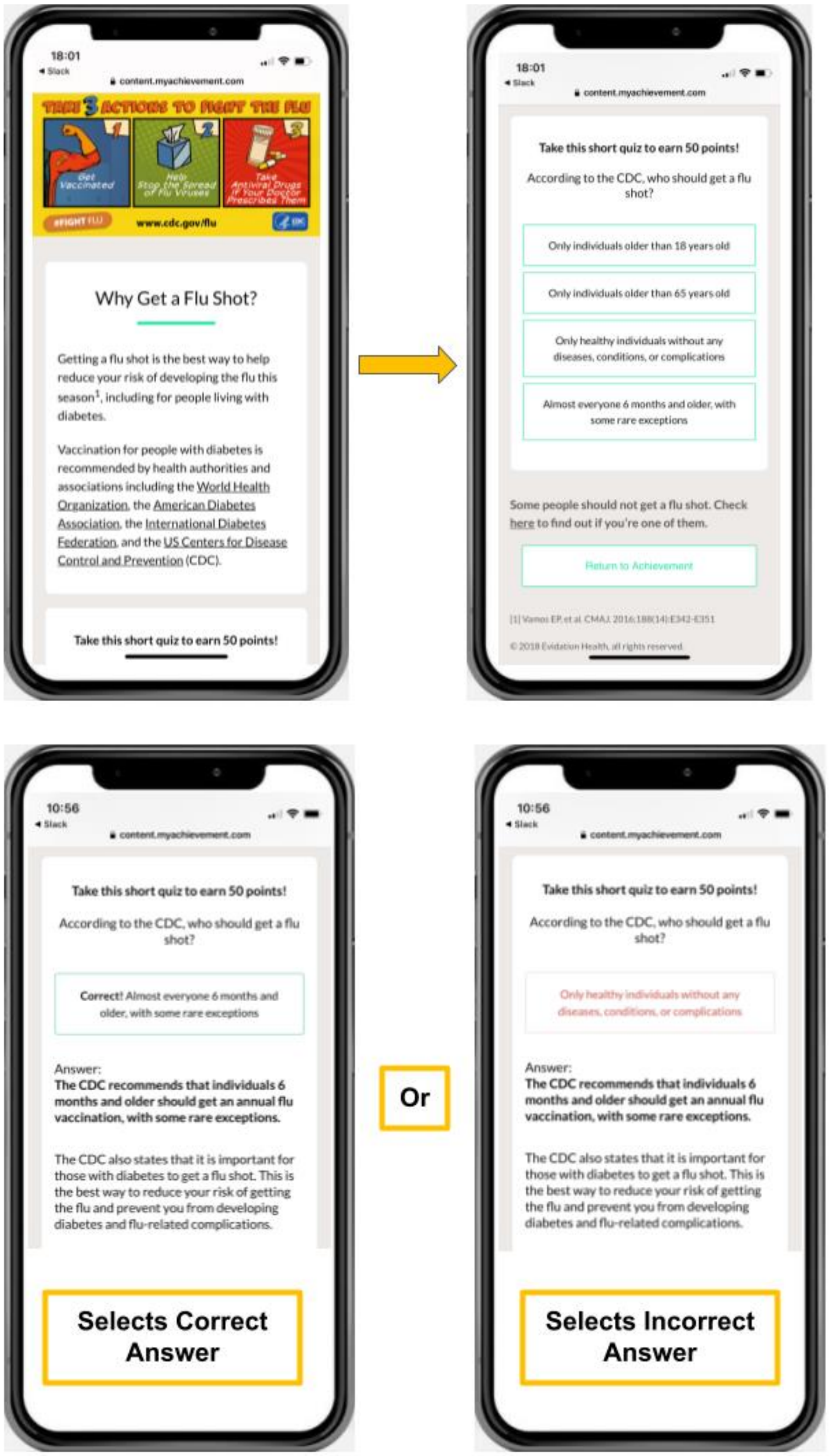

b) Third message:

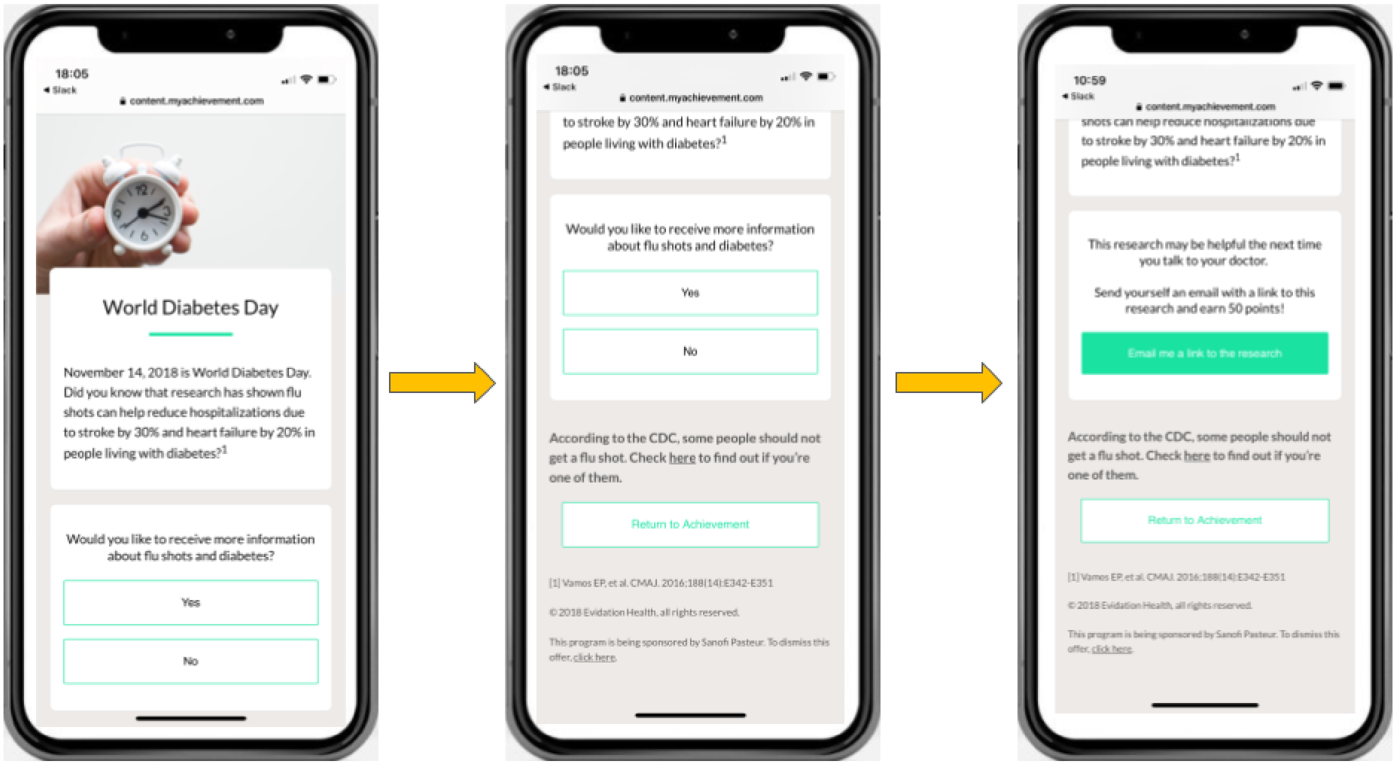

c) Fifth message:

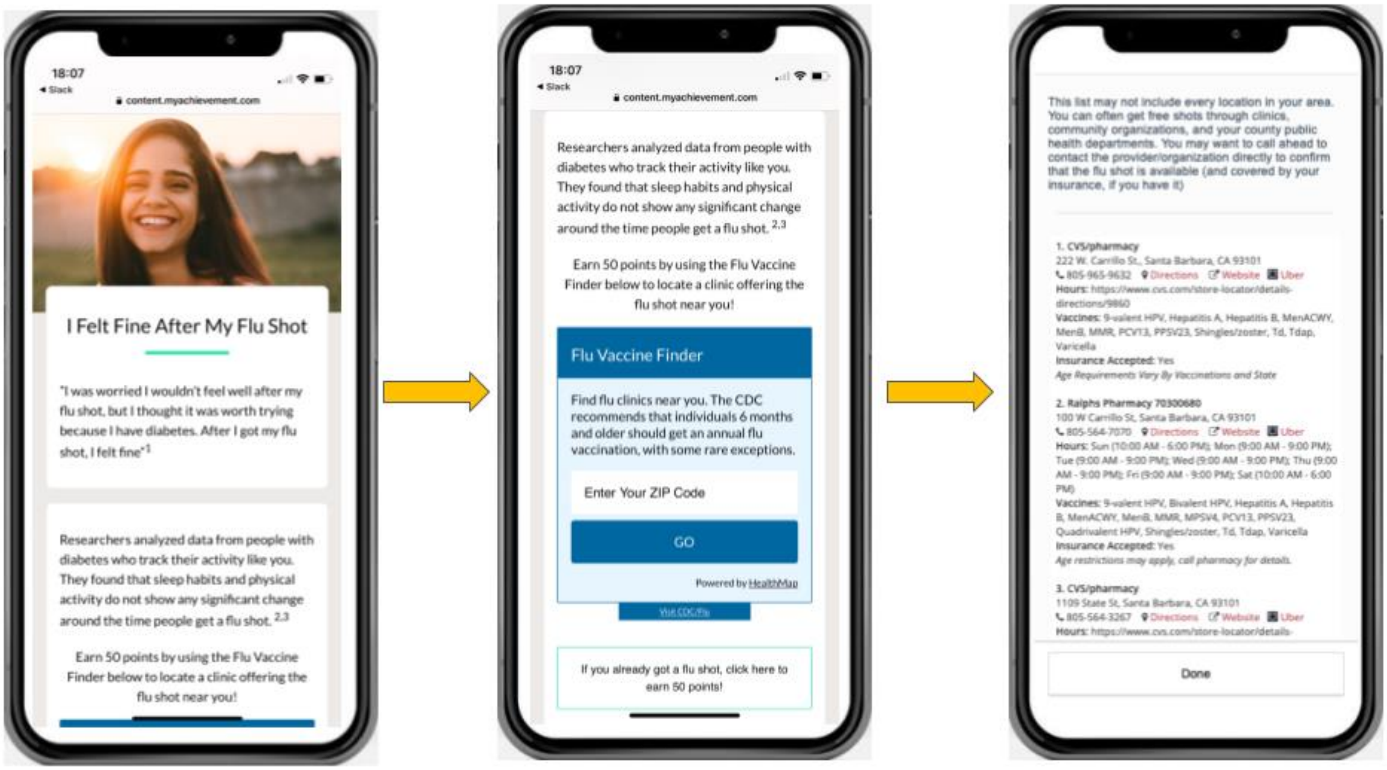

Supplement: Supplementary file 1 — Supplementary Information [file 41746_2021_508_MOESM1_ESM.pdf]
