# Supplementary material for: Digital intervention increases influenza vaccination rates for people with diabetes in a decentralized randomized trial
Source: NPJ Digit Med. 2021 Sep 17;4:138. doi: 10.1038/s41746-021-00508-2 (PMC8448887; doi:10.1038/s41746-021-00508-2)
Supplement: Supplementary file 2 — Reporting Summary [file 41746_2021_508_MOESM2_ESM.pdf]

nature research

Corresponding author(s): Sandrine Samson

Last updated by author(s): 08/10/2021

Reporting Summary

Nature Research wishes to improve the reproducibility of the work that we publish. This form provides structure for consistency and transparency in reporting. For further information on Nature Research policies, see our Editorial Policies and the Editorial Policy Checklist.

Statistics

For all statistical analyses, confirm that the following items are present in the figure legend, table legend, main text, or Methods section.

n/a	Confirmed	
☐	☒	The exact sample size (n) for each experimental group/condition, given as a discrete number and unit of measurement
☐	☒	A statement on whether measurements were taken from distinct samples or whether the same sample was measured repeatedly
☐	☒	The statistical test(s) used AND whether they are one- or two-sided<br>*Only common tests should be described solely by name; describe more complex techniques in the Methods section.*
☐	☒	A description of all covariates tested
☐	☒	A description of any assumptions or corrections, such as tests of normality and adjustment for multiple comparisons
☐	☒	A full description of the statistical parameters including central tendency (e.g. means) or other basic estimates (e.g. regression coefficient) AND variation (e.g. standard deviation) or associated estimates of uncertainty (e.g. confidence intervals)
☐	☒	For null hypothesis testing, the test statistic (e.g. F, t, r) with confidence intervals, effect sizes, degrees of freedom and P value noted<br>*Give P values as exact values whenever suitable.*
☒	☐	For Bayesian analysis, information on the choice of priors and Markov chain Monte Carlo settings
☐	☒	For hierarchical and complex designs, identification of the appropriate level for tests and full reporting of outcomes
☐	☒	Estimates of effect sizes (e.g. Cohen's d, Pearson's r), indicating how they were calculated

Our web collection on statistics for biologists contains articles on many of the points above.

Software and code

Policy information about availability of computer code

Data collection	No software was used
Data analysis	Qualified researchers may request access to the aggregate results and related study documents including the study report, study protocol with any amendments, blank case report form, statistical analysis plan, and dataset specifications. Further details on Sanofi's data sharing criteria, eligible studies, and process for requesting access can be found at https://www.clinicalstudydatarequest.com.

For manuscripts utilizing custom algorithms or software that are central to the research but not yet described in published literature, software must be made available to editors and reviewers. We strongly encourage code deposition in a community repository (e.g. GitHub). See the Nature Research guidelines for submitting code & software for further information.

Data

Policy information about availability of data

All manuscripts must include a data availability statement. This statement should provide the following information, where applicable:

- Accession codes, unique identifiers, or web links for publicly available datasets
- A list of figures that have associated raw data
- A description of any restrictions on data availability

Qualified researchers may request access to the aggregate results and related study documents including the study report, study protocol with any amendments, blank case report form, statistical analysis plan, and dataset specifications. Further details on Sanofi's data sharing criteria, eligible studies, and process for requesting access can be found at https://www.clinicalstudydatarequest.com.

Field-specific reporting

Please select the one below that is the best fit for your research. If you are not sure, read the appropriate sections before making your selection.

☐ Life sciences ☒ Behavioural & social sciences ☐ Ecological, evolutionary & environmental sciences

For a reference copy of the document with all sections, see nature.com/documents/nr-reporting-summary-flat.pdf

Behavioural & social sciences study design

All studies must disclose on these points even when the disclosure is negative.

Study description	Decentralized randomized pragmatic trial
Research sample	Adults with self-reported diabetes in the Achievement app (myachievement.com.) were selected for inclusion in the trial: Intervention group - M age (SD) = 46.8 (11·1), 78·5% female; Control group - M age (SD) = 46.7 (11·2), 79·4% female). Participants were chosen from the Achievement app, as the intervention was delivered within the app. The participants were predominantly female, White, and in the middle age range and had high levels of income and education.
Sampling strategy	Simple random sampling based upon an a priori list of participants to intervention and control groups was used. For sample size determination, we estimated a 2·7% increase in vaccination rate between the intervention and control groups. This value was selected to be consistent with prior research and clinically meaningful. Power analysis indicated the need for an analysis set of 4,043 individuals in each arm of the study (total N = 8,086) to achieve 80% power to detect a 2·7% increase in vaccination rate with a type I error rate of 0·05 (25). To account for potential non-response to study surveys, we tagged 31,404 people with diabetes for study inclusion, with 15,702 randomized to each of the two arms (~25% assumed response rate).
Data collection	Participants were sent an online baseline questionnaire via link in an email, a mid-study assessment at three months, and a final assessment at six months. Due to the study design, participants could complete any or all assessments; completion of mid-study and final assessment was not predicated on completion of the baseline assessment. The primary endpoint of influenza vaccination status was collected in the three- and/or six-month questionnaires. Questions on demographics, influenza vaccination status, and healthcare worker recommendation were asked of all participants. Participants in the intervention cohort were additionally asked about their perceptions of the interventions, and their engagement with intervention tasks was assessed.
Timing	Launched in September of 2018 and the last participant completed the final survey in April of 2019
Data exclusions	A total of 101 individuals reported discrepant answers at the 3-month and 6-month questionnaires with regard to vaccination status. The effect size remained unchanged and statistically significant after re-running the primary outcome without these individuals.
Non-participation	Approximately one-third of participants who were enrolled and randomized reported on the final endpoint at either 3 or 6 months of the study. This was consistent with expectations, given that participants were blinded to study participation in this pragmatic randomized controlled trial.
Randomization	Stratified randomization based upon sex and age as covariates was used to randomize intervention and control groups.

Reporting for specific materials, systems and methods

We require information from authors about some types of materials, experimental systems and methods used in many studies. Here, indicate whether each material, system or method listed is relevant to your study. If you are not sure if a list item applies to your research, read the appropriate section before selecting a response.

Materials & experimental systems

n/a	Involved in the study	
☒	☐	Antibodies
☒	☐	Eukaryotic cell lines
☒	☐	Palaeontology and archaeology
☒	☐	Animals and other organisms
☐	☒	Human research participants
☐	☒	Clinical data
☒	☐	Dual use research of concern

Methods

n/a	Involved in the study	
☒	☐	ChIP-seq
☒	☐	Flow cytometry
☒	☐	MRI-based neuroimaging

Human research participants

Policy information about studies involving human research participants

Population characteristics	See above

Recruitment	Participants were selected from a pool of individuals who self-reported having diabetes within the Achievement App, developed by Evidation Health. They were randomized and blinded to study enrollment, as well as treatment arm (intervention or control).
Ethics oversight	Study was approved by Solutions Institutional Review Board (Little Rock, AR)

Note that full information on the approval of the study protocol must also be provided in the manuscript.

Clinical data

Policy information about clinical studies

All manuscripts should comply with the ICMJE guidelines for publication of clinical research and a completed CONSORT checklist must be included with all submissions.

Clinical trial registration	clinicaltrials.gov: NCT03870997
Study protocol	Qualified researchers may request access to the aggregate results and related study documents including the study report, study protocol with any amendments, blank case report form, statistical analysis plan, and dataset specifications. Further details on Sanofi's data sharing criteria, eligible studies, and process for requesting access can be found at https://www.clinicalstudydatarequest.com.
Data collection	Online data collection, between September 2018 and April 2019.
Outcomes	Primary outcome was self-reported vaccination status and was reported on clinicaltrials.gov prior to analysis
